# Prepregnancy Maternal Weight and Body Mass Index of Children with and without Congenital Heart Disease

**Published:** 2014-06

**Authors:** Mehdi Ghaderian, Abdol-Rahman Emami-Moghadam, Mohammad-Reza Khalilian, Kourosh Riahi, Fatemeh Ghaedi

**Affiliations:** 1 Department of Pediatric Cardiology, Emam Hosein Medical, Educational and Research Center, Esfahan University of Medical Sciences; 2 Department of Pediatric Cardiology; 3 Pediatric Endocrinology; 4 Golestan Hospital, Jundishapur University of Medical Sciences, Ahvaz, Iran

**Keywords:** Congenital Heart Defects; Maternal Obesity; Maternal Overweight

## Abstract

***Objective:*** Congenital heart diseases are among the most frequent major congenital anomalies. One of the suspected reasons for congenital heart defects is overweight and obesity of mother during prepregnancy and pregnancy. We studied the relationship between maternal overweight and obesity and the risk of congenital anomaly.

***Methods:*** All of children with congenital heart defect (164 infants with major nonsyndromic heart disease) referred to our pediatric cardiology clinic or admitted to our ward during 2011-2012 were included in this study. Controls were 158 live-born infants without any major malformations and their mothers. Mothers of these infants were studied for weight, height and body mass index (BMI).

***Findings*** ***:*** The most frequent congenital heart disease was ventricular septal defect (39%), patent ductus arteriosus (11%), complete atrioventricular septal defect (10%), pulmonary stenosis (9.1%), and atrial septal defect (8.5%). There was no significant difference between maternal age (*P*=0.1), weight (*P*=0.8) and height (*P*=0.3) in the two groups. The mothers had not significantly higher BMI than that of the control mothers. Compared with underweight (BMI <18.5) and normal weight women (OR: 1.24, 95%CI: 0.40-3.89), overweight (OR: 0.98, 95%CI: 0.31-3.10) and obese women (OR: 1.16, 95%CI: 0.34-4.00) were not more likely to have an infant with a congenital heart defect.

***Conclusion:*** This study suggests that there may not be a relation between maternal BMI and having a child with congenital heart defect.

## Introduction

Congenital heart diseases, which affect at least one percent of newborns, are among the most frequent major congenital anomalies and responsible for excess morbidity, premature death, health-care costs and mortality^[^^1^^,^^2^^]^. It has been difficult to find the cause of congenital heart defects, and many identified causes (for example, rubella, diabetes mellitus, retinoic acid, and infections) are uncommon in many populations. Obesity and overweight have been associated with pregnancy complications and adverse reproductive outcomes.

 Overweight (body mass index [BMI] >25-30 kg/m^2^, calculated as weight in kilograms divided by height in meters squared) and obesity (BMI≥30 kg/m^2^) are major global and especially in developed countries, public health and economic concern. According to estimates overall prevalence of obesity in women aged between 20 to 39 years in the United States had increased from 15% in 1976-1980 to 34% in 2008^[^^3^^-^^5^^]^. Obesity and overweight also appear correlated inversely to educational level of women and it seems to have highest rate among women with educational level less than high school^[^^6^^]^. Worldwide, in 2005 it was estimated that 1.6 billion adults (older than 15 years) were overweight, and 400 million were obese^[^^7^^]^. By 2015, it is supposed that there will be more than 2.5 billion overweight and 700 million obese adults in the world. One third of women in the United States aged older than 15 years were obese in 2004^[^^8^^]^. 

 Moghimi-Dehkordi et al reported that in Iran the overall prevalence of overweight and obesity was 34.1% and 15.4% and that prevalence of overweight and obesity is moderately high in the general Iranian population^[^^9^^]^. For both mother and her child there are significant health complications and adverse reproductive outcomes of prepregnancy maternal obesity. For the mother, these may include gestational diabetes, type 2 diabetes, systemic hypertension, eclampsia and preeclampsia, thromboembolic disorders, increased deep vein thrombosis, endothelial dysfunction and is an independent predictor of coronary artery disease and premature death in adults and increased cesarean delivery rates. Children of these mothers are at increased risk of birth problems, macrosomia, overweight, and increased perinatal death of fetus^[^^10^^,^^11^^]^. Development of congenital anomalies may also be associated with maternal obesity and overweight. Some studies have shown that obese women have an increased risk for certain types of congenital heart disease^[^^12^^-^^15^^]^. Congenital anomalies are one of the leading causes of stillbirth and infant mortality. 

 We studied the relationship between maternal overweight and obesity and the risk of congenital anomaly in their infant comparing it with controls.

## Subjects and Methods

During 2011-2012 all of children with congenital heart defect referred to our pediatric cardiology clinic or admitted to our ward in Golestan Medical, Educational, and Research Center, Jundishapur University of Medical Sciences, Ahvaz, Iran were included in this study. Inclusion criterion for children was having an isolated or complex congenital heart disease established by echo-cardiography, angiography or other appropriate methods. Exclusion criteria for children were having family history of congenital heart disease, eclampsia, exposure to cardiac teratogenes, substance abuse or radiation.

 The study group was classified according to the main cardiac defect and each subject was assigned a principal cardiac diagnosis. The group included all congenital cardiac defects and controls were live-born infants without any major malformations and their mothers. Study group and controls were frequency matched by region of birth, place of residence and age.

 Mothers of both groups completed a questionnaire regarding their status of health, medical care received, fertility and pregnancy history, nutrition and vitamin intake in the 3 months before conception through the first 3 months of pregnancy, occupational and environmental exposures, substance abuse and family history. Those with gestational diabetes or diabetes before pregnancy or if their child had a genetic syndrome, chromosomal defect or a syndromic heart defect, were excluded from the study. Initially 16 infants with syndromic congenital heart disease were excluded. During the study 8 infants in study group and 5 control infants whose mothers reported having been diagnosed with diabetes mellitus or gestational diabetes were further excluded. So, 158 infants without birth defects (controls) and 164 infants with major nonsyndromic heart disease (study group) were studied. Demographic data such as weight and height were gathered from medical records and other data extracted from questionnaires. Underweight was defined as a BMI <18.5 kg/m^2^, and normal weight as a BMI between 18.5 and 25 kg/m^2^, overweight as a BMI >25 and 29.9 kg/m^2^, obesity as a BMI ≥30 kg/m^2^. 

 So, 158 infants without birth defects (controls) and 164 infants with major nonsyndromic heart disease (study group) were studied. Demographic data such as weight and height were gathered from medical records and other data extracted from questionnaires. Underweight was defined as a BMI <18.5 kg/m^2^, and normal weight as a BMI >8.5<25 kg/m^2^, overweight as a BMI >25 and 29.9 kg/m^2^, obesity as a BMI ≥30 kg/m^2^. 

 The SPSS version 18 statistical software was used for all statistical analyses. All data are expressed as mean±standard deviation (SD) and 95% confidence interval. Odds ratios (ORs) and 95% confidence intervals (CIs) were calculated to compare obese or overweight mothers with those with underweight or normal weight. Demographic data of the tudy group and controls were compared by using chi-square test. Patients were divided by major type of congenital heart disease and patients with multiple diseases formed one group. Statistical significance was defined as *P*< 0.05.

***Findings***

There were 164 children with congenital heart disease (study group) and 158 healthy children as controls available for our study. Table 1 shows the frequency of infants according to the major type of congenital heart disease. The most frequent diagnoses were ventricular septal defect (39%), patent ductus arteriosus (11%), complete atrioventricular septal defect (10%), pulmonary stenosis (9.1%), and atrial septal defect (8.5%). Acyanotic heart disease is more frequent (116 patients, 70%) than cyanotic heart disease in our patients. 

 Demographic data of mothers and children is shown in Table 2 and compared between the two groups and their mothers. There was no significant difference between maternal age, weight and height in the two groups. The mothers of defect children had not significantly higher BMIs than the control mothers.

 Compared with underweight (BMI <18.5) and normal weight women (OR: 1.24, 95%CI: 0.40-3.89), overweight (OR: 0.98, 95%CI: 0.31-3.10) and obese women (OR: 1.16, 95%CI: 0.34-4.00) were not more likely to have an infant with a congenital heart defect. 

 Overweight and obesity were not significantly associated with congenital heart defects in our analysis. When all congenital heart defects were considered together, obesity was not a significant risk factor. Examination of the data for effects of weight and infant sex showed no significant relation in analyses.

 Women, who were overweight or obese, were not at increased risk of having an infant with congenital heart defects.

## Discussion

Congenital heart diseases are among the most frequent major congenital anomalies and responsible for excess morbidity, premature death, health-care costs and mortality. 

**Table 1:** Frequency of patients according to major types of congenital heart disease

**Diagnoses**	**n**	**%**
**Ventricular Septal Defect**	64	39
**Atrial Septal Defect**	14	8.5
**Patent Ductus Arteriosus**	18	11
**Aortic** **Stenosis**	1	0.6
**Pulmonary Stenosis**	15	9.1
**Coarctation of Aorta**	4	2.4
**Complete Atrioventricular Septal Defect**	16	10
**Tetralogy of Fallot**	10	6.2
**Transposition of the Great Arteries**	2	1.2
**Tricuspid atresia**	2	1.2
**Pulmonary atresia**	2	1.2
**Single ventricle**	2	1.2
**Others**	14	8.5
**Total**	164	100

**Table 2:** Demographic data of the study group and controls

**Parameter**		**Study group**	**Controls**	***P-*** **value**
**Maternal age**		27.29 (5.65)	26.34 (5.42)	0.1
**Mother weight (kg)**		63.15 (12.59)	63.44 (11.96)	0.8
**Mother height (cm)**		157.34 (5.86)	158.08 (5.96)	0.3
**Body Mass Index ** **(Kg/m** ^2)^	**Underweight (<18.5)**	8 (4%)	10 (6%)	0.6
	**normal weight (18.5–25)**	70 (43%)	74 (47%)	0.9
	**Overweight (>25-29.9)**	63 (39%)	52 (33%)	0.8
	**Obese (>30)**	23 (14%)	22 (14%)	0.8
**Infant data**	**Birth weight (infant) (kg)**	3.05 (0.6)	3.12 (5.7)	0.2
	**Weight (children) (kg)**	10.5 (0.9)	10.8 (1.0)	0.3
	**Age (month)**	26.7 (33.7)	27.7 (39.3)	0.4

It has been difficult to find causes of congenital heart defects. Obesity and overweight have been associated with pregnancy complications and adverse reproductive outcomes and were suggested as being increased risk for congenital heart defects.

 Reasons for the potential relation between elevated prepregnancy weight in mothers and congenital heart defects in infants are unknown. Previous studies expressed different opinions and are often difficult to compare. Although one study reported no increased risk for conotruncal heart defects, other studies reported risk elevations for transposition of the great arteries and defects of the great vessels and truncus arteriosus^[^^12^^,^^15^^,^^16^^]^. Waller et al described an elevated OR for transposition of the great arteries and great arteries defects but not for defects of septal closure^[^^12^^]^. Queisser-Luft et al in their study reported elevated ORs among mothers with BMI above 30 for some heart anomalies including truncus arteriosus and transposition of the great arteries^[^^15^^]^. Recent meta-analysis findings described that, overweight women were not at a significantly increased risk of all congenital heart defects as a group or of individual defects^[^^14^^]^. We did not find an elevated risk of congenital heart defects among mothers with normal weight, overweight, or obese women compared with underweight mothers. Our study suggests that there may not be a correlation between maternal BMI and having a child with a congenital heart defect. This study, although small, did not confirm the previously observed elevated risk for congenital heart defects in overweight or obese women.

 The mechanism for the association between obesity and birth defects is not definitely known, but several possible factors have been described and proposed^[^^12^^,^^17^^]^. In a recent study, Giboa et al did not find a significant (the 95% CI did not exclude 1) increase in the OR in women whose BMI was between 30 and 35 for all congenital heart defects as in our study, but they did find an increase in overweight women in their study^[^^18^^]^.

 Another known risk factor for birth defects is that women who are obese might have diabetes^[^^19^^,^^20^^]^. Diabetes mellitus is associated with increased rates of congenital heart defects thought to be caused by different mechanisms. Both obesity and diabetes cause multiple metabolic problems. It is suggested that by more than one mechanism the obesity can produce a wide range of abnormalities in carbohydrate and lipid metabolism, insulin resistance, and adipocyte hormone action and may cause congenital defects. Early biological measurements such as insulin levels and glucose levels during routine medical care or early in pregnancy would be informative in determining hyperglycemia, hyperinsulinemia, or some other metabolic abnormalities for detection of high risk pregnancies. 

 In our study we tried to decrease the bias, the findings may still show some unknown amount of underlying confound. For example, we excluded women who reported any type of diabetes and gestational diabetes from our study; however, our study may still have obese mothers with undiagnosed diabetes.

 Our study suggests that there may not be a correlation between maternal BMI and having a child with a congenital heart defect. Our study, although small, did not confirm the previously observed elevated risk for congenital heart defects.

 Another described theory is that overweight and obese women might have increased requirement for vitamins (e.g. folic acid) known to be protective against congenital heart defects. Multivitamin and folic acid intake is similar among obese and non-obese women; and if these women had increased requirement of these micronutrients they would have had increased risk for congenital heart defects. Women with increased BMI may be more likely to be nutritionally deficient because of a diet restriction and intake of foods. In obese women the protective effect of folic acid in reducing the risk of neural tube defect may not be observed^[^^21^^]^. It is suggested that a common underlying etiology for congenital defects such as genetic factors could be responsible for these defects. Deficiencies in other micro-nutrients and vitamins may be associated with other congenital anomalies. There is no discussed reason for the reduced OR of congenital heart disease in underweight women. Underweight women may have a lower need for vitamins and micronutrients such as folate, associated with risk of congenital heart defects, than do overweight, or obese women; or they might also have more nutrient-rich diets during the prepregnancy and periconceptional period than heavier women.

 In our country because of decreasing pregnancy rate and increasing health care, especially for pregnant women, nearly all pregnant mothers receive multivitamins and folic acid during prepregnancy and pregnancy period and we do not think that women in our country have deficiency of vitamins that influence birth defects. 

 In the past, ultrasound examination was more difficult in obese women, potentially resulting in less diagnosis and terminations of pregnancy for fetal cardiac anomaly and therefore increased prevalence of these defects at birth. By increasing experience of operators and using newer equipments and paraclinical study, evaluation and termination of high risk pregnancies are done easier in high risk mothers, and we think that many women bearing a child with a congenital heart defect, had aborted or terminated pregnancy, without informing us, so that we could not take their data and BMI into our statistics. 

 This study was population based. Small number of samples limited our ability to assess the relation between overweight and obesity and each of congenital heart defects. In our study we tried to exclude women with any type of diabetes and other genetic disorders but underlying disorders without clinical symptoms and diagnosis could not be excluded which would influence the study results. We could not exclude environmental factors such as chemicals. We assume interaction of different factors causing congenital defects in fetus. By increasing health care of pregnant women and using vitamins especially folic acid and decreasing pregnancy and child birth, it is suggested that underlying disorders play more prominent role in occurring congenital defects in pregnancies, especially in apparently healthy women. By increasing age of pregnancy underlying disorders especially genetic disorders also could influence fetal genetics and congenital defects. Other studies with larger sample size could elucidate this. 

 One limitation of this study was the small size of samples, so that it was not possible to compare subgroups and different congenital heart defects. The small size of study also limited our data to evaluate the relation between specific heart defects and maternal BMI. Another limitation was that we could not examine mothers directly and our data are taken from medical records and these may be inaccurate partly in measuring or recording of data. As congenital heart defects are the most common cause of mortality during first months of birth many of these children were not included in our study and we had no data of them and their mothers, this could also be a limitation to our study.

## Conclusion

This study suggests that there may not be a relation between maternal BMI and having a child with congenital heart defect. By increasing overweight and obesity in population and previous suggestion for influence of these factors in congenital anomalies and especially heart defects, more studies with larger sample size are needed to compare other factors such as underlying disorders. Temporarily, to reduce weight seems to be a better suggestion. 

## Authors Contribution

M. Ghaderian: Design, Study Supervision, Critical Revision of the Manuscript, Data Analysis, Interpretation

A. Emami-Moghadam: Drafting of the Manuscript

M.R. Khalilian: Drafting of the Manuscript

K. Riahi: Drafting of the Manuscript, Interpretation

F. Ghaedi: Acquisition of Data, Drafting of the Manuscript
